# A stacked learning framework for accurate classification of polycystic ovary syndrome with advanced data balancing and feature selection techniques

**DOI:** 10.3389/fphys.2025.1435036

**Published:** 2025-05-06

**Authors:** Heba M. Emara, Walid El-Shafai, Naglaa F. Soliman, Abeer D. Algarni, Reem Alkanhel, Fathi E. Abd El-Samie

**Affiliations:** ^1^ Department of Electronics and Electrical Communications Engineering, Ministry of Higher Education Pyramids Higher Institute (PHI) for Engineering and Technology, 6th of October City, Egypt; ^2^ Automated Systems and Soft Computing Lab (ASSCL), Computer Science Department, Prince Sultan University, Riyadh, Saudi Arabia; ^3^ Department of Information Technology, College of Computer and Information Sciences, Princess Nourah bint Abdulrahman University, Riyadh, Saudi Arabia

**Keywords:** ADAYSN, boruta, cervical cancer, data balancing, feature selection, machine learning, pcos, stacked learning

## Abstract

**Introduction:**

In the domain of women’s health, the intricate conditions of Polycystic Ovary Syndrome (PCOS) demand sophisticated methodologies for accurate identification and intervention.

**Methods:**

This study introduces an innovative machine learning framework tailored to precisely classify instances of PCOS. The methodology incorporates stacked learning and depends on the Adaptive Synthetic (ADASYN) algorithm, Synthetic Minority Over-sampling Technique (SMOTE), and random oversampling methods for addressing data imbalances. The BORUTA technique is used for feature selection, with the overarching objective of advancing precision and performance metrics in classification tasks.

**Results:**

Within the scope of PCOS classification, the proposed framework achieves a commendable 97% accuracy. These results underscore the proficiency of the proposed framework in discriminating PCOS cases with a high degree of precision. Critical to this contribution is the rigorous comparative analysis against existing methodologies, affirming the superior accuracy and performance attributes of the proposed framework.

**Discussion:**

This substantiates its potential as a transformative tool in medical classification. Moreover, beyond immediate applications, this paper explores the generalization of the proposed framework, demonstrating its adaptability and efficacy across different medical classifications. This versatility is exemplified by its successful application to cervical cancer, showcasing the framework potential as a pioneering force in reshaping the landscape of machine-learning applications in healthcare diagnostics.

## 1 Introduction

Cervical cancer and Poly-Cystic Ovary Syndrome (PCOS) are two significant health issues that greatly impact women’s health worldwide. Cervical cancer is the fourth most common cancer among women, and early detection is crucial to improving survival rates (Terasawa et al., 2022). Similarly, PCOS, a common endocrine disorder affecting fertility and metabolic health, requires timely diagnosis to prevent long-term health risks (Norman et al., 2024). Globally, PCOS affects approximately 6%–10% of women of reproductive age (Johnstone et al., 2010), with the prevalence in the United States estimated to be as high as 12%–18% (March et al., 2010; Goodman et al., 2015).

Despite advancements in diagnostic methods, women in underdeveloped regions face significant barriers in accessing reliable diagnostic tools, such as the Pap smear, which is the gold standard for cervical cancer detection (Saslow et al., 2012; Crum et al., 2018). In these regions, the lack of access to essential screening methods has led to higher mortality rates due to delayed diagnosis and treatment. Even in areas where Pap smears are available, the test has limitations, including lower sensitivity, which can result in false negatives for detecting pre-cancerous cells (Wiley et al., 2004).

The situation is equally concerning for PCOS, as the condition presents with non-specific symptoms that often complicate early diagnosis. This further highlights the need for innovative diagnostic approaches that can enhance accuracy and accessibility, especially in low-resource settings (McCartney and Marshall, 2016; Barber et al., 2007). By improving early detection of both cervical cancer and PCOS, there is an opportunity to significantly improve women’s health outcomes globally.

Machine learning (ML) has shown great promise in addressing these challenges by providing more accurate, efficient, and accessible diagnostic tools. Al Mudawi and Alazeb (2022) proposed a comprehensive approach for cervical cancer prediction using classic ML classifiers such as Random Forest (RF), Decision Tree (DT), and Support Vector Machine (SVM). They reported an impressive 100% accuracy, demonstrating the potential of ML in the medical domain. In another study, Tak et al. (2022) applied machine learning to predict the outcomes of Hinselmann, Schiller, cytology, and biopsy tests for cervical cancer. Their study achieved a high accuracy of 97.5% using a Fine Gaussian SVM for Hinselmann classification. However, both studies faced challenges related to class imbalance, which can bias predictions toward the majority class (negative cases) and reduce the effectiveness of the model for minority classes.

Addressing class imbalance has become a focal point in cervical cancer diagnosis using ML techniques. Kuruvilla and Jayanthi (2023) proposed an Ensemble Feature Selection (EFS) approach combined with SMOTE (Synthetic Minority Over-sampling Technique) to handle class imbalance and improve diagnostic accuracy across multiple tests, including Hinselmann, Schiller, cytology, and biopsy. Their approach achieved accuracy values of over 94% for these diagnostic methods. Furthermore, Le Ngoc and Huyen (2023) explored the use of deep learning techniques with the Keras framework, incorporating class weighting and oversampling to improve cervical cancer detection, achieving a 94.18% accuracy. Despite these advancements, challenges such as overfitting and dataset generalization remain, especially in imbalanced datasets.

In addition to cervical cancer, ML techniques have also been applied to improve the diagnosis of PCOS. Aggarwal et al. (2023) used statistical feature selection methods, including Chi-Square, ANOVA, and Mutual Information (MI), to enhance the prediction of PCOS, achieving a 93.52% accuracy with a Random Forest classifier. Bharati et al. (2020) employed univariate feature selection and reported an accuracy of 91.01% with an RFLR classifier. Although these studies have made significant strides in improving PCOS diagnosis, they are often limited by the use of small, domain-specific datasets, which reduce the generalizability of the models.

Furthermore, research on cervical cancer diagnosis using more advanced deep learning and feature selection methods is expanding. Ashok and Aruna (2016) explored feature selection methods for cervical cancer detection using SVM classifiers. Their approach involved image pre-processing, multi-thresholding techniques, and shape and textural feature extraction, leading to a classification accuracy of 98.5%. Plissiti et al. (2018) used a Convolutional Neural Network (CNN) architecture with 3×3 filters and 2×2 max-pooling to classify cervical cancer based on cytomorphological features, achieving superior performance compared to Multi-Layer Perceptron (MLP) and SVM classifiers. Another deep learning method proposed by Kuruvilla and Jayanthi (2023) integrated the EFS with feature optimization techniques like Entropy Elephant Herding Optimization (EEHO) and Entropy-based Butterfly Optimization (EBFO), achieving high accuracy for multiple cervical cancer tests.

While these models have demonstrated high accuracy in cervical cancer diagnosis, there remains a need for improved techniques to integrate feature selection and handle class imbalance simultaneously. This paper proposes a novel approach to improve the classification of both PCOS and cervical cancer by combining a stacked ensemble framework with advanced machine learning algorithms. The proposed framework addresses class imbalance by incorporating the ADAYSN algorithm, which generates synthetic samples to balance the dataset and reduce bias toward the majority class. Additionally, the BORUTA feature selection method is employed to identify the most relevant features, enhancing model interpretability and reducing computational complexity.

The contributions of this paper are as follows:

• A stacked ensemble framework that improves classification accuracy for both PCOS and cervical cancer compared to existing methods.
• The integration of the ADAYSN algorithm to effectively handle class imbalance, leading to more accurate detection of rare conditions such as cervical cancer.
• The use of the BORUTA feature selection method to enhance feature relevance, reduce dimensionality, and improve model interpretability.

The rest of this paper is structured as follows. Section 2 presents a detailed review of the dataset and explains the proposed methodology, focusing on the stacked ensemble framework, ADAYSN algorithm, and BORUTA feature selection. Section 3 provides the results and compares the proposed framework with state-of-the-art methods. Finally, Section 4 concludes with a summary of the findings and the potential future applications of the proposed approach.

## 2 Materials and methods

### 2.1 Data description

The dataset utilized in this study to examine cervical cancer risk factors was obtained from the University of California, Irvine (UCI) Machine Learning Repository (ICS, 2024). It was collected at Hospital Universitario de Caracas in Caracas, Venezuela, specifically in 2019, and it comprises demographic data, lifestyle choices, and previous medical records of 858 patients. For PCOS analysis, the dataset was acquired from the Kaggle Dataset Repository (Kaggle, 2023), containing information from 10 different Indian hospitals and encompassing data for 541 women. The dataset consists of 43 features, including physical attributes, hormone levels (Luteinizing Hormone (LH), Follicle-Stimulating Hormone (FSH), First Beta-Human Chorionic Gonadotropin (I beta-HCG), Second Beta-Human Chorionic Gonadotropin (II beta-HCG), Thyroid-Stimulating Hormone (TSH), Anti-Müllerian Hormone (AMH), Prolactin (PRL), Progesterone (PRG), Random Blood Sugar (RBS)), and other medical indicators. These features are used to classify women as either diagnosed with PCOS (177 instances) or not diagnosed (364 instances). Both datasets provide critical information for analyzing cervical cancer and PCOS risk factors. Descriptions of each dataset’s features can be found in Tables 1, 2.

**TABLE 1:** Cervical cancer dataset description.

No.	Attribute	No. of missing values	No.	Attribute	No. Of missing values
1	Age	0	19	STDs: pelvic inflammatory disease	105
2	Number of sexual partners	26	20	STDs: genital herpes	105
3	First sexual intercourse	7	21	STDs: molluscum contagiosum	105
4	Num of pregnancies	56	22	STDs: acquired immunodeficiency syndrome (AIDS)	105
5	Smokes	13	23	STDs: human immunodeficiency virus (HIV)	105
6	Smokes (years)	13	24	STDs: hepatitis B	105
7	Smokes (packs/year)	13	25	STDs: human papillomavirus (HPV)	105
8	Hormonal contraceptives	108	26	STDs: number of diagnoses	0
9	Hormonal contraceptives (years)	108	27	STDs: time since first diagnosis	787
10	IUD (intrauterine device)	117	28	STDs: time since last diagnosis	787
11	Sexually transmitted diseases (STDs)	105	29	Dx: cancer	0
12	STDs (number)	105	30	Dx: cervical intra-epithelial (CIN)	0
13	STDs: condylomatosis	105	31	Dx: HPV	0
14	STDs: cervical condylomatosis	105	32	Dx	0
15	STDs: vaginal condylomatosis	105	33	Hinselmann	0
16	STDs: vulvo-perineal condylomatosis	105	34	Schiller	0
17	STDs: syphilis	105	35	Cytology	0
18	STDs: pelvic inflammatory disease	105	36	Biopsy	0

**TABLE 2:** PCOS dataset description.

No.	Feature	No.	Feature	No.	Feature
1	PCOS class label	15	I beta-HCG	29	Weight gain
2	Age	16	II beta-HCG	30	Hair growth
3	Weight	17	FSH	31	Skin darkening
4	Height	18	LH	32	Hair loss
5	BMI	19	FSH/LH	33	Pimples
6	Blood Group	20	Hip (inch)	34	Fast food
7	Pulse rate	21	Waist (inch)	35	Regular Exercise
8	RR (breaths/min)	22	Hip Ratio	36	BP Systolic
9	Hb	23	TSH	37	BP Diastolic
10	Cycle	24	AMH	38	Follicle No. (L)
11	Cycle length (days)	25	PRL	39	Follicle No. (R)
12	Marriage Status	26	Vitamin D3	40	Avg. F size (L)
13	Pregnant	27	PRG	41	Avg. F size (R)
14	No. of abortions	28	RBS	42	Endometrium

### 2.2 Evaluation of datasets

Both datasets have significant challenges due to missing values, small size, and class imbalance. Addressing these issues is crucial for building robust classification models. In the cervical cancer dataset, several attributes contain missing values. For example, attributes related to sexually transmitted diseases (STDs) have over 100 missing values, while two attributes have 787 missing values. These missing values can introduce biases and reduce the predictive power of the model if not properly addressed. Similarly, in the PCOS dataset, certain features such as Marriage Status, beta-HCG, AMH, and Fast Food had missing values. During data preparation, the missing values in these features were filled using the median value of the corresponding instances.

The cervical cancer dataset is relatively small, with only 858 samples, and is severely imbalanced. Only 2.1% of the patients (18 individuals) were diagnosed with cervical cancer, leading to a significant class imbalance that could bias the model towards the majority class (non-cancer cases). The PCOS dataset faces a similar challenge. Out of 541 women, only 177 were diagnosed with PCOS, leading to an imbalance between the two classes (PCOS and non-PCOS). Addressing this imbalance is crucial to avoid a model biased towards the majority class. To address these challenges, we employed data balancing techniques such as SMOTE and ADASYN, which generate synthetic samples to balance the dataset and improve the model ability to predict minority class outcomes. These preprocessing steps are essential for achieving robust and accurate classification results.

### 2.3 Theoretical background

#### 2.3.1 Data balance techniques

In order to address the issue of data imbalance in our dataset, we employed three data balance techniques: SMOTE, Radom over-sampling and ADAYSN.

1. Synthetic Minority Over-sampling Technique: SMOTE is popular for addressing class imbalance in machine learning datasets (Sowjanya and Mrudula, 2023). When dealing with imbalanced datasets, where the number of instances in the minority class is much smaller than that in the majority class, traditional classifiers may perform poorly, as they work in favor of the majority class due to its higher frequency (Demir and Şahin, 2022). SMOTE is designed to alleviate this issue by generating synthetic samples for the minority class, thus balancing the class distribution (Fernández et al., 2018). Given a dataset with the minority class represented by $X_{\text{min}}$ and the majority class represented by $X_{\text{maj}}$ , SMOTE proceeds as follows (Chawla et al., 2002):
  (a) Choose a minority class instance, denoted as $x_{i}$ , from $X_{\text{min}}$ .
  (b) Select k nearest neighbors of $x_{i}$ from $X_{\text{min}}$ using a distance metric, such as Euclidean distance.
  (c) Randomly select one of the k nearest neighbors, denoted as $x_{\text{nn}}$ .
  (d) Generate a synthetic instance, denoted as $x_{\text{new}}$ , by interpolating between $x_{i}$ and $x_{\text{nn}}$ using Equation 1:
$$x_{\text{new}}=x_{i}+\text{rand}\left(0,1\right)\times \left(x_{\text{nn}}-x_{i}\right),$$ (1)
    where rand(0,1) is a random number between 0 and 1.
  (e) Repeat steps 1-4 to generate a desired number of synthetic samples for the minority class.
  (f) Append the synthetic samples to the original minority class instances, resulting in a balanced dataset.
2. Adaptive Synthetic Sampling: ADASYN (He et al., 2008) is an extension of the SMOTE algorithm that addresses the limitations of SMOTE in handling imbalanced datasets with overlapping classes. ADASYN adaptively generates synthetic samples based on the level of difficulty in learning the minority class instances. The ADASYN algorithm starts by determining the number of synthetic samples to be generated for each minority class instance based on the ratio of the difference between the number of its k-nearest neighbors from the majority and the minority class instances. The minority class instance is $x_{i}$ , and its set of k nearest neighbors is $N_{i}$ . The number of synthetic samples to be generated for $x_{i}$ is calculated using Equation 2:

$$G_{i}=\frac{\left(\left|N_{i}|-|N_{i}\cap \text{minority class}\right|\right)\times \text{desired\_ratio}}{\left|N_{i}\cap \text{minority class}\right|}$$ (2)

where desired_ratio is the desired balance ratio of the number of instances between the majority and minority classes. Next, for each minority class instance $x_{i}$ , ADASYN generates $G_{i}$ synthetic samples. Similar to SMOTE, a random neighbor $x_{n}$ is chosen from $N_{i}$ , and a synthetic instance $x_{\text{new}}$ is created using Equation 3:

$$x_{\text{new}}=x_{i}+\lambda \times \left(x_{n}-x_{i}\right)$$ (3)

where 0<λ<1 . The ADASYN algorithm iterates through all minority class instances, generating synthetic samples adaptively according to the difficulty of learning the instances. This adaptivity allows ADASYN to focus on the regions of the minority class that are harder to learn, providing a more effective approach for handling imbalanced datasets with overlapping classes. ADASYN has been widely used in various classification tasks and has demonstrated improvements in performance compared to SMOTE and other traditional over-sampling techniques.

#### 2.3.2 BORUTA feature selection

The BORUTA algorithm focuses on identifying the most relevant features for classification tasks. Given a dataset X with n instances and p features, BORUTA aims to determine the importance of each feature with respect to the target variable y (Kursa and Rudnicki, 2010).

• RF Feature Importance Calculation: BORUTA depends on an RF classifier to calculate feature importance scores. For each feature j , the algorithm constructs shadow features by shuffling the values of feature j , while preserving the relationships between features. The dataset with the original features and shadow features is used to train the RF classifier, which yields importance scores for each feature (refers to Equation 4).

$$\text{Imp}\left(j\right)=\text{Importance score of feature }j$$ (4)

• Feature Selection: BORUTA compares the importance scores of the original features with those of their shadow features. Features with higher importance scores than the maximum importance score of their shadow features are considered relevant and retained for further analysis (refers to Equation 5).

$${\text{MaxImp}}_{\text{shadow}}=\underset{\text{shadow features}}{\max}\left(\text{Imp}\left(j\right)\right)$$ (5)

Features with $\text{Imp}\left(j\right)>{\text{MaxImp}}_{\text{shadow}}$ are selected as relevant.

• Iterative Process: The feature selection process is repeated until BORUTA identifies the relevant features with high confidence. The algorithm evaluates the importance scores in each iteration and stops when the difference between the maximum importance score of the original features and the maximum importance score of their shadow features is not statistically significant. The selected relevant features can then be used to build accurate classification models for the target variable y .

The proposed algorithm focuses on cervical cancer and PCOS classification using the BORUTA feature selection technique and ensemble learning based on correlation work. BORUTA employs an RF classifier to identify the most relevant features for classification tasks, rather than minimizing feature sets for specific models. It undergoes iterative steps to determine the important features. For cervical cancer classification with a dataset of 36 features, BORUTA outputs important features based on their importance scores, such as Feature1, Feature5, Feature10, and Feature20. Similarly, for PCOS classification with a dataset of 42 features, BORUTA suggests significant features like Feature2, Feature8, Feature15, and Feature30. The iterative evaluation of feature importance and statistical tests allows BORUTA to pinpoint the most relevant features for cervical cancer and PCOS classification. These selected features are then used to train classification models, such as RF and SVM, to predict the presence or absence of cervical cancer or PCOS. BORUTA feature selection algorithm offers a systematic and robust approach for identifying relevant features in cervical cancer and PCOS classification. By being model-independent in feature importance assessment, it facilitates the development of accurate classification models for medical conditions.

#### 2.3.3 Stacked learning

Stacked learning, also known as stacked generalization or stacking, is an ensemble learning technique that combines the predictions of multiple base classifiers to make more accurate and robust predictions. The idea behind stacked learning is to leverage the diverse perspectives of individual classifiers by training them on the same dataset and then using their predictions as input features for a higher-level meta-classifier. This meta-classifier learns to combine the base classifiers predictions and generate the final prediction (Mohamed et al., 2023).

The advantages of stacked learning are significant. Firstly, it often leads to improved predictive performance compared to using a single classifier. By aggregating the predictions from multiple base classifiers, stacked learning can capture complex patterns and relationships that may be missed by individual classifiers. It benefits from the ensemble effect, where the errors made by individual classifiers can be mitigated when combined. This reduction in bias and variance leads to more accurate and reliable predictions.

Secondly, stacked learning enhances the robustness of the classification model. Since it relies on the consensus of multiple base classifiers, stacked learning is less susceptible to the biases and errors of individual classifiers. Outliers or noise in the data that may disproportionately affect a single classifier are attenuated by the ensemble approach, resulting in more robust predictions that generalize well to unseen data.

In the proposed structure with LR, RF, and KNN as base classifiers and XGBoost as the meta-classifier, each component serves a crucial role. LR, RF, and KNN are selected as base classifiers due to their distinct modeling approaches and strengths. LR, a linear classifier, can capture linear relationships and provide interpretable coefficients. RF, an ensemble of decision trees, excels at handling complex interactions and nonlinear relationships. KNN, a non-parametric classifier, relies on nearest neighbors to make predictions, and can capture local patterns, effectively. By combining these diverse classifiers, the ensemble model benefits from their complementary strengths and can capture a broader range of patterns and relationships in the data.

XGBoost is chosen as the meta-classifier for several reasons. XGBoost is a gradient boosting algorithm known for its high performance and ability to handle complex relationships. It effectively learns from the meta-features generated by the base classifiers and makes accurate predictions. Additionally, XGBoost offers flexibility in hyperparameter tuning, enabling further optimization of the ensemble model. Its boosting capabilities amplify the strengths of the base classifiers and improve the overall predictive performance of the stacked model.

Overall, the proposed structure leverages the power of stacked learning by combining LR, RF, and KNN as base classifiers and utilizing XGBoost as the meta-classifier. This ensemble-based approach benefits from the diversity and flexibility of the base classifiers, while harnessing the boosting capabilities of XGBoost to create a powerful classification model with improved accuracy and robustness.

Table 3 showcases the classifiers used in the code, along with their respective parameters, definitions, and example values. This tabular representation highlights the significance of optimizing parameter values through the grid search algorithm. The obtained parameter values are essential as they have been carefully selected to enhance the performance of each classifier. By conducting an extensive search over various combinations, the grid search algorithm identifies the best parameter configuration for each classifier. These optimized values are crucial for achieving improved classification accuracy and ensuring that the classifiers are appropriately calibrated for the specific dataset and classification task at hand. Therefore, the provided table not only serves as a reference for the parameter values but also emphasizes the importance of parameter optimization through grid search, ultimately contributing to more effective and reliable classification outcomes.

**TABLE 3:** Hyperparameters for the proposed machine learning classifiers.

Classifier	Parameter	Definition	Value
LR	random_state	Controls the random seed for reproducibility	42
	max_iter	Maximum number of iterations for convergence	1,000
	C	Inverse of regularization strength smaller values specify stronger regularization	1.0
RF	n_estimators	Number of trees in the forest	100
	random_state	Controls the random seed for reproducibility	42
	max_depth	Maximum depth of the trees	10
	min_samples_split	Minimum number of samples required to split an internal node	2
KNN	n_neighbors	Number of neighbors to consider for classification	5
XgBoost	learning_rate	Step size shrinkage to prevent overfitting	0.1
	max_depth	Maximum depth of a tree	3
	n_estimators	Number of boosted trees	100
	subsample	Subsample ratio of the training instances	0.8

### 2.4 Proposed approach

**FIGURE 1:**
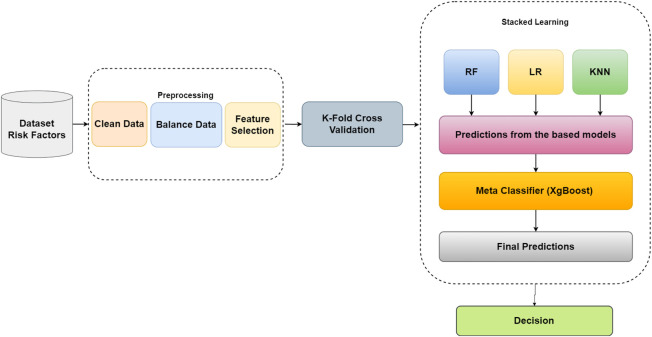
Block diagram of the proposed approach.

Figure 1 illustrates the proposed framework, which involves pre-processing steps to ensure data suitability for classification. Initially, redundant features are removed through a feature selection process, and categorical variables are transformed into numerical representations. The imputation technique addresses missing values, ensuring data completeness. Subsequently, BORUTA feature selection is applied to identify the most relevant features, reducing dataset dimensionality and focusing on informative features for classification. To address class imbalance, over-sampling techniques are employed, creating a balanced dataset split into training and testing sets. Stacked learning is utilized with base classifiers (LR, RF, and KNN) to generate meta-features that capture collective knowledge. These meta-features, along with the target variable, train a meta-classifier (XGBoost), which is optimized using grid search cross-validation. The trained meta-classifier is used to predict the target variable for the testing set. Evaluation metrics, such as ROC curve, AUC score, and confusion matrix, are used to assess the model performance. By integrating pre-processing, feature selection, and stacked learning with a meta-classifier, the proposed framework achieves an enhanced classification accuracy, providing more reliable predictions. The algorithm commences with a pre-processed dataset, StackedFeatures, and corresponding labels, y, as inputs for binary classification. The dataset is split into training and testing sets (80% and 20%, respectively). Base models (LR, KNN, RF) and the meta model (XGBoost) are initialized. The BORUTA feature selection algorithm identifies relevant features based on their importance scores. Stacking learning is performed, and meta-features are created by combining base model predictions on the training and testing sets. The meta-model, XGBoost, is trained on the stacked features, StackedTrainX. Predictions on the stacked test features, StackedTestX, are made using the trained meta-model. The algorithm outputs the predicted labels for the test instances, allowing for the classification of unseen data. Throughout the algorithm, various variables, such as StackedFeatures, y, StackedTrainX, StackedTestX, and selected features, represent the data and computations during the execution. The proposed algorithm, as depicted in Algorithm 1, aims to enhance classification performance through a combination of BORUTA feature selection and stacking learning with base models (LR, KNN, RF) and a meta model (XGBoost). The algorithm starts by taking the pre-processed dataset, StackedFeatures , and corresponding labels, y , as input. To evaluate the model performance, the dataset is split into training and testing sets using the TrainTestSplit function, resulting in TrainX , TestX , Trainy , and Testy . The base models, LR, KNN, and RF, and the meta model, XGBoost, are initialized, setting the foundation for the subsequent feature selection and stacking learning steps.

The algorithm proceeds with feature selection using the BORUTA algorithm. The base models (LR, KNN, RF) are iteratively trained on TrainX , and their feature importance scores are obtained. The BORUTA algorithm then selects relevant features based on the comparison of feature importance scores, ensuring the retention of the most informative features. Following feature selection, stacking learning is applied. The base models (LR, KNN, RF) are trained on the selected features from TrainX . The predictions of these base models on both TrainX and TestX are concatenated, creating StackedTrainX and StackedTestX , respectively. Next, the meta model, XGBoost, is trained on StackedTrainX . This meta-classifier learns from the base models’ predictions and combines their knowledge to make more accurate predictions on unseen instances. Finally, the algorithm employs the trained meta model to predict the target labels for StackedTestX . providing the predicted labels for the test instances.

The output of the algorithm comprises the predicted labels for the test instances, allowing for the classification of unseen data.

Algorithm 1 BORUTA Feature Selection and Stacking Learning with LR, KNN, RF, and XGBoost.

**Input**: Preprocessed dataset StackedFeatures , Labels y

**Output**: Predicted labels for test instances TrainX , TestX , Trainy , Testy← TrainTestSplit ( StackedFeatures , y ); //Split data into training and test sets
**Initialization**:
Initialize base models: LR, KNN, RF
Initialize meta model: XGBoost
**Feature Selection using BORUTA**:
**while** *BORUTA feature selection not converged* **do**
 Train base models LR, KNN, RF on TrainX

 Obtain feature importance scores from base models;
 Apply BORUTA algorithm to select relevant features;
**end**
**Stacking Learning**:
Train base models LR, KNN, RF on selected features;
Obtain stacked features StackedTrainX , StackedTestX by
concatenating predictions of base models on TrainX , TestX

Train meta model XGBoost on StackedTrainX

**Prediction**:
Make predictions on StackedTestX

**return** Predicted labels for TestX

### 2.5 Performance metrics

The number of correct estimates from all predictions is used to determine if a model is successful when it is developed from scratch or when it is employed in place of an existing model. But this data simply reveals if the classification was accurate. The classification accuracy alone is typically insufficient to assess a model suitability. The confusion matrix is used to describe the estimated outputs of a classifier. The classification model performance with a set of known test data is typically described using Table 4 with 4 parameters called a confusion matrix. False positives $F_{p}$ , false negatives $F_{n}$ , true positives $T_{p}$ , and true negatives $T_{n}$ are their names. The performance metrics are presented in Table 5.

**TABLE 4:** Confusion matrix: actual vs. Predicted classes.

		Predicted	
		Positive	Negative
Actual	Positive	$T_{p}$	$F_{n}$
	Negative	$F_{p}$	$T_{n}$

**TABLE 5:** Calculation formulas and explanations of performance metrics.

Measure	Formula	Evaluation focus
Accuracy	$\frac{T_{p}+T_{n}}{T_{p}+T_{n}+F_{p}+F_{n}}$	It is used to calculate the ratio of the number of correctly estimated samples to all samples If the model utilized is highly accurate, it might be regarded as the best
Precision	$\frac{T_{p}}{T_{p}+F_{p}}$	The proportion of positively identified samples that were accurately predicted to be positive samples
Recall	$\frac{T_{p}}{T_{p}+F_{n}}$	It is used to calculate the percentage of positive values that are considered to be true
F1-Score	$2*\frac{Precision\times Recall}{Precision+Recall}$	It is the sensitivity harmonic mean. Consequently it considers both false positives and false negatives

## 3 Results

This section presents the outcomes of the classification models applied to two healthcare domains: PCOS and Cervical Cancer classification.

### 3.1 Results for PCOS classification

The initial phase of the study involved applying four distinct machine learning algorithms (RF, KNN, LR, and XGBoost) to the dataset to assess each model’s predictive capabilities for PCOS classification. The stacked learning technique was then employed, combining predictions from multiple models, which significantly improved classification accuracy compared to individual models.

To address the challenge of data imbalance, the ADASYN algorithm was applied, balancing the skewed class distributions. This created a more representative training set, enhancing the model’s ability to classify PCOS cases. Additionally, BORUTA feature selection was integrated with stacked learning to identify the most important features, leading to a reduced dataset size and improved interpretability. This approach not only streamlined the model but also made its decision-making process more transparent, aiding clinical application.

#### 3.1.1 PCOS classification using machine learning models

**FIGURE 2:**
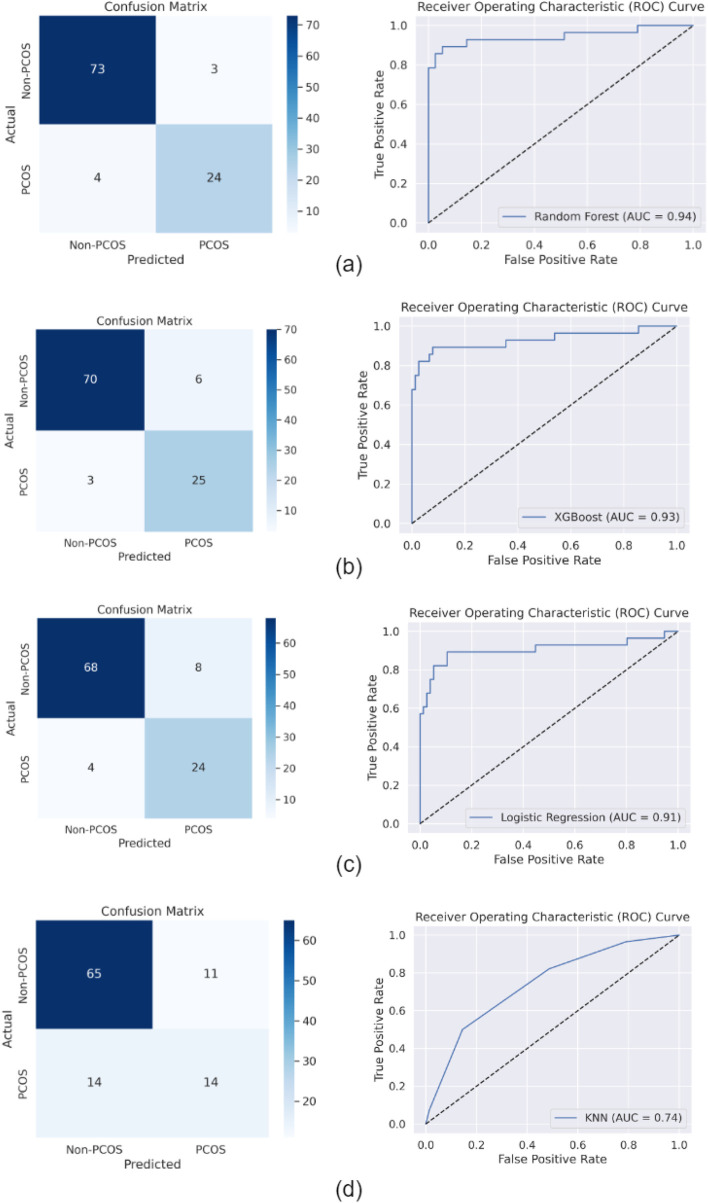
Confusion matrices and ROC curves for machine learning models: **(A)** RF, **(B)** XGBoost, **(C)** LR, and **(D)** KNN.

Four machine learning models were used for PCOS classification: RF, KNN, LR, and XGBoost. These models were selected due to their ability to handle complex classification tasks. Figure 2 displays the confusion matrices and ROC curves for each model.

Table 6 summarizes the performance metrics, including accuracy, recall, precision, and F1 score, for each model.

**TABLE 6:** Performance metrics for machine learning models on PCOS classification.

Model	Accuracy (%)	Recall (%)	Precision (%)	F1 score (%)
RF	92	92	92	92
XGBoost	91	91	92	91
LR	88	88	89	89
KNN	76	76	75	76

RF and XGBoost achieved the highest accuracy (92% and 91%), while KNN had the lowest (76%). Both RF and XGBoost also demonstrated strong recall (92% and 91%), with LR following at 88%. In terms of precision, RF and LR performed best (92% and 89).

#### 3.1.2 Results for stacked learning model

The performance of the stacked learning model was evaluated using 5-fold cross-validation. This technique divides the dataset into five subsets, with each subset being used as a validation set once, while the others are used for training. The results across the folds were averaged to obtain robust performance metrics, such as Precision, Recall, F1-score, and Support, which provide a thorough understanding of the model’s performance across different partitions.

**FIGURE 3:**
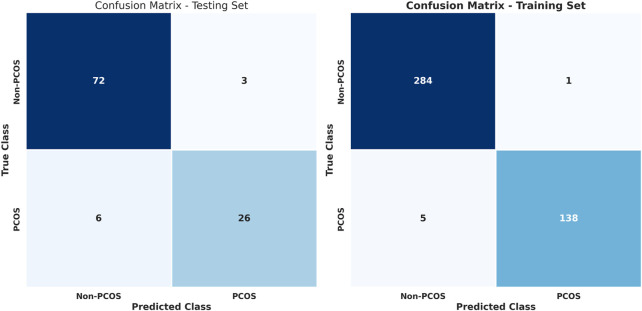
Confusion matrices for training and testing using stacked learning with imbalanced data.

Figure 3 illustrates confusion matrices for training and testing using the stacked learning model with imbalanced data. Table 7 presents the classification results, showing that in the testing set, non-PCOS instances achieved a Precision of 0.92, Recall of 0.96, and F1-score of 0.94, while PCOS cases exhibited a Precision of 0.90, Recall of 0.81, and F1-score of 0.85. The overall accuracy was 0.92.

**TABLE 7:** Classification report for training and testing using stacked learning on imbalanced data for PCOS classification.

	Testing set				Training set			
	Precision	Recall	F1-score	Support	Precision	Recall	F1-score	Support
Non-PCOS	0.92	0.96	0.94	75	0.98	1.00	0.99	285
PCOS	0.90	0.81	0.85	32	0.99	0.97	0.98	143
Accuracy	0.92				0.99			
Macro avg	0.91	0.89	0.90	107	0.99	0.98	0.98	428
Weighted avg	0.92	0.92	0.91	107	0.99	0.99	0.99	428

The training set results demonstrated higher performance, with non-PCOS showing a Precision of 0.98, Recall of 1.00, and F1-score of 0.99, while PCOS attained Precision of 0.99, Recall of 0.97, and F1-score of 0.98, achieving an overall accuracy of 0.99. Despite these results, the model struggled to identify PCOS cases in the testing set, indicating a challenge with data imbalance.

**FIGURE 4:**
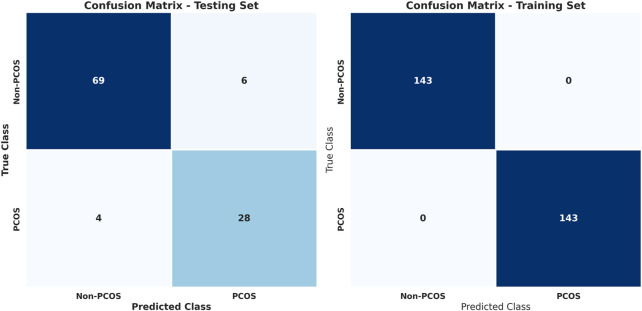
Confusion matrices for training and testing using stacked learning combined with random undersampling for PCOS classification.

To address the issue of imbalanced data, various techniques such as SMOTE, ADASYN, and random undersampling were employed. These methods adjusted the class distribution within the training data to create a more balanced representation. Figure 4 and Table 8 show the results for stacked learning combined with random undersampling.

Table 9 compares various methods for PCOS classification. The proposed ADASYN-based approaches show significant improvements in feature selection and performance. ADASYN + Boruta reduces the feature count to just 9, while achieving a competitive accuracy of 97%, compared to Danaei et al.‘s 33 features and 98.89% accuracy. The proposed methods demonstrate strong recall, particularly with ADASYN achieving a recall of 98%, matching the best in the comparison. ADASYN effectively addresses class imbalance, and when combined with Boruta, improves model interpretability while maintaining high performance. Overall, the proposed approaches outperform traditional methods in both efficiency and accuracy, emphasizing the value of advanced oversampling and feature selection techniques.

**TABLE 8:** Classification report for training and testing using stacked learning with random undersampling for PCOS classification.

	Testing set				Training set				
	Precision	Recall	F1-score	Support	Precision	Recall	F1-score	Support	
Non-PCOS	0.95	0.92	0.93	75	Non-PCOS	1.00	1.00	1.00	143
PCOS	0.82	0.88	0.85	32	PCOS	1.00	1.00	1.00	143
Accuracy	0.91				Accuracy	1.00			
Macro avg	0.88	0.90	0.89	107	Macro avg	1.00	1.00	1.00	286
Weighted avg	0.91	0.91	0.91	107	Weighted avg	1.00	1.00	1.00	286

**TABLE 9:** Comparison of the proposed classification approach for PCOS detection with state-of-the-art methods.

Authors	Classifier	Technique	Selected features	Performance measure
Denny et al. (2019)	RF	PCA	23	Accuracy of 89.02%
Bharati et al. (2020)	RFLR	Univariate feature selection	10	Accuracy of 91.01% and Sensitivity of 90%
Nandipati et al. (2020)	RF	SMOTE	41	Accuracy of 93.12%
Aggarwal et al. (2023)	RF	ANOVA	11	Accuracy of 93.52%
Munjal et al. (2020)	RF	GA	9	Accuracy of 83% and Sensitivity of 64%
Tiwari et al. (2022)	KNN and SVM	-	10	Accuracy of 93.25%
Tanwani (2020)	LR	Filter-based (correlation)	10	Accuracy of 92% and Sensitivity of 93%
Neto et al. (2021)	RF	-	-	Accuracy of 95% and Sensitivity of 94%
Inan et al. (2021)	XGBoost	Chi-Square and ANOVA	23	Accuracy of 96.03% and Sensitivity of 98%
Danaei Mehr and Polat (2022)	RF	Pearson filter	33	Accuracy of 98.89% and Sensitivity of 100%
Proposed Approach	Stacked Learning	ADASYN	43	Accuracy of 98% and Recall of 98%
		ADASYN + Boruta	9	Accuracy of 97% and Recall of 96%

### 3.2 Results for cervical cancer detection

The models that demonstrated superior performance in PCOS detection, including the Stacked Learning model with ADASYN and BORUTA, were applied to the cervical cancer classification task. The primary aim was to evaluate the effectiveness of these models in detecting cervical cancer and to assess their generalizability across different medical conditions.

The cervical cancer dataset presented a significant class imbalance. Out of the total instances, only 18 samples belonged to the cancer class, while 840 instances were classified as no-cancer. This severe imbalance posed a challenge for accurate classification, as models could be biased toward predicting the majority class. To address this issue, the ADASYN algorithm was employed to oversample the minority class. As a result, the cancer class was balanced to 837 samples, closely matching the no-cancer class with 840 samples. This adjustment mitigated the imbalance, allowing for more effective model training and evaluation.

**FIGURE 5:**
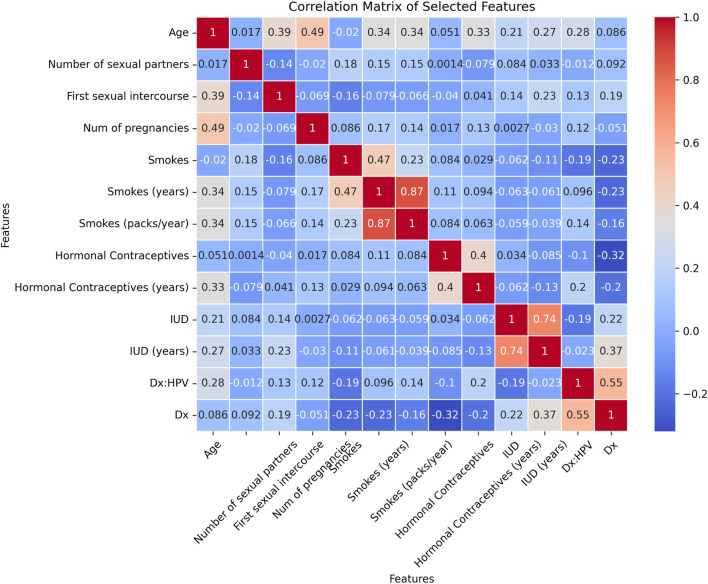
Heatmap for the selected features using BORUTA algorithm for cervical cancer classification.

Following data balancing, the BORUTA feature selection algorithm was used to identify the most relevant features for classification. Out of the initial 36 features, 13 were identified as important for the cervical cancer detection task, as shown in the heatmap in Figure 5. These features include Age, Number of sexual partners, First sexual intercourse, Num of pregnancies, Smokes, Smokes (years), Smokes (packs/year), Hormonal Contraceptives, Hormonal Contraceptives (years), IUD, IUD (years), Dx: HPV, and Dx. The heatmap highlights the correlations between these selected features and the target variable, offering insights into the key factors influencing cervical cancer detection.

**FIGURE 6:**
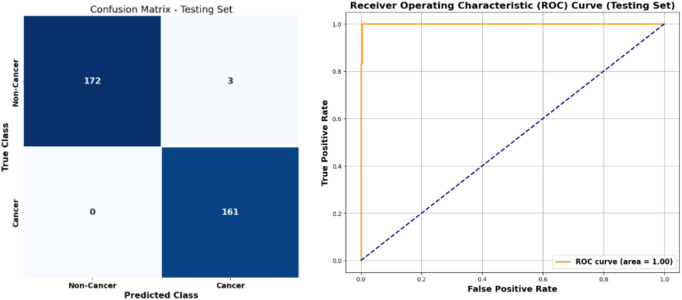
Confusion matrix and ROC curve of stacked learning model combined with ADASYN and BORUTA algorithms for cervical cancer classification.

The performance of the stacked learning model combined with ADASYN and BORUTA is illustrated in Figure 6, which shows the confusion matrix and ROC curve. The model achieved a high accuracy in detecting both cancer and no-cancer cases, with true positives $\left(T_{p}\right)$ of 172 and true negatives $\left(T_{n}\right)$ of 161. The low number of false positives $\left(F_{p}\right)$ at 3 indicates the model’s ability to minimize misclassifications, while the absence of false negatives $\left(F_{n}\right)$ reflects its success in detecting all cancer cases in the dataset. The AUC of 1.00 further underscores the model’s excellent performance and its capacity to differentiate between cancer and no-cancer instances.

In summary, the results for cervical cancer classification using the proposed framework demonstrate its effectiveness and generalizability. With an accuracy of 99%, recall of 98%, precision of 100%, and an F1-score of 99%, the model performs exceptionally well in identifying both cancer and non-cancer cases. The recall of 98% reflects the model’s ability to correctly identify the majority of cancer cases, while the precision of 100% indicates its capability to avoid false positive predictions. These results highlight the robustness and reliability of the proposed framework, making it a promising tool for real-world applications in cervical cancer detection.

Table 10 presents a comparative analysis of the proposed classification framework for cervical cancer detection alongside state-of-the-art methods. The proposed framework, utilizing Stacked Learning, ADASYN, and BORUTA, achieves an accuracy of 99%, outperforming most other models listed in terms of accuracy and reliability. The superior accuracy of the proposed framework highlights its effectiveness in distinguishing between cervical cancer and non-cancer cases, making it a highly accurate tool for medical diagnosis. The framework’s recall of 98% and precision of 100% further emphasize its ability to minimize both false negatives and false positives, which is critical in medical decision-making. Compared to other methods, which show a range of accuracies from 68% to 99.3%, the proposed framework excels by consistently demonstrating robustness and generalizability across different datasets. This suggests its potential applicability in various clinical settings and populations, offering a reliable solution for cervical cancer detection.

**TABLE 10:** Comparison of the proposed classification framework for cervical cancer detection with state-of-the-art methods.

Authors	Technique	Dataset	Total images/Attributes	Performance measure
Al Mudawi and Alazeb (2022)	PMS + SVM + KNN	UCI dataset	32 attributes	SVM: 99% accuracy
	Logit model + Deep learning	UCI dataset	36 attributes	94.18% accuracy
William et al. (2018)	SVM	Real dataset (150 images)	150	Accuracy 98.5%, Sensitivity 98%, Specificity 97.5%
Sompawong et al. (2019)	Enhanced fuzzy c means	Herlev dataset	917	95.00% accuracy, 100% sensitivity, 90.00% specificity
William et al. (2019)	autoencoders and softmax	Real dataset	-	Accuracy 97.8%
Adem et al. (2024)	GoogleNet	Herlev dataset	917	Accuracy 94.5%
Lin et al. (2019)	CNN	Herlev dataset	917	68.0% accuracy
Gorantla et al. (2019)	KNN, SVM, DT, RF, XGBoost, CNN	Herlev dataset	917	Accuracy 93%
Yilmaz et al. (2009)	CNN-SVM	Real dataset	2000	Accuracy 99.3%, Sensitivity 98.9%, Specificity 99.4%
Kano et al. (2021)	2D UNet and 3D UNet	Real dataset	98 cases	Not mentioned
Mohammed et al. (2021)	CNN	SIPaKMeD	4,049	Accuracy of 99%
Xu et al. (2020)	KNN, SVM, DT	Herlev dataset	917 Public dataset	Accuracy 99.27%
Proposed Approach	Stacked Learning + ADAYSN + BORUTA	UCI	13	Accuracy of 99%, recall of 98% and precision of 100%

## 4 Discussion

### 4.1 PCOS detection

The performance of various machine learning models for PCOS classification was thoroughly evaluated in this study. As shown in Table 6, RF and XGBoost models performed significantly better in terms of accuracy, recall, precision, and F1-score compared to LR and KNN. RF and XGB achieved accuracies of 92% and 91% respectively, indicating their superior ability to predict PCOS cases. Additionally, the F1-score, which balances precision and recall, shows that RF (92%) and XGB (91%) outperform LR and KNN, which have lower F1-scores of 89% and 76%, respectively.

The introduction of stacked learning further enhanced the model performance, particularly in addressing the issue of class imbalance. Utilizing the ADASYN (Adaptive Synthetic Sampling) algorithm for oversampling minority classes improved the predictive ability of the models, as evidenced by the improved recall (81%) for the PCOS class, and an overall accuracy of 92%. Despite this, the model still exhibited a lower performance in predicting PCOS instances on the testing set, indicating potential challenges related to the class imbalance, even with the use of ADASYN. Incorporating BORUTA for feature selection further improved model interpretability and reduced overfitting, allowing the model to focus on the most relevant features for PCOS detection.

The results of different data balancing techniques, such as Random Undersampling, SMOTE, and ADASYN, demonstrated that ADASYN consistently produced the best results, as evidenced by the confusion matrix and classification reports. The oversampling strategy enabled the model to better learn and classify minority instances without losing significant information from the majority class.

The study reveals that RF, XGB, and SL models combined with ADASYN and BORUTA algorithms offer robust performance in classifying PCOS. Despite the challenges posed by class imbalance, the application of data balancing techniques and feature selection significantly improved classification accuracy and model generalizability.

### 4.2 Cervical cancer detection

The proposed framework was also applied to the cervical cancer classification task, and the results were equally promising. The dataset exhibited a severe class imbalance, with only 18 instances of cervical cancer compared to 840 instances of non-cancer. To address this imbalance, ADASYN was used to oversample the minority class, resulting in a balanced dataset that allowed for effective training and evaluation of the models.

The stacked learning model, combined with ADASYN and BORUTA, performed exceptionally well, achieving an accuracy of 99%, a recall of 98%, and a precision of 100%. These results underscore the effectiveness of the proposed framework in accurately detecting cervical cancer, even in cases where the class distribution is highly skewed. The confusion matrix in Figure 6 illustrates the model’s ability to correctly classify both cancer and non-cancer instances with minimal false positives and no false negatives, resulting in a high area under the curve (AUC) score of 100. This high performance is critical in clinical settings where both precision and recall are of utmost importance for ensuring accurate diagnosis and treatment planning.

Furthermore, BORUTA feature selection played a key role in reducing the feature set from 36 to 13 relevant attributes. These features were highly correlated with the target variable and included significant clinical parameters such as age, number of pregnancies, smoking history, and human papillomavirus (HPV) diagnosis. The correlation heatmap in Figure 5 visually demonstrates the strength of these associations, providing a clear understanding of the factors contributing to cervical cancer detection.

In comparison to state-of-the-art methods, as summarized in Table 10, the proposed framework consistently outperformed existing approaches. While previous studies reported accuracies ranging from 68% to 99.3%, the proposed framework achieved an unparalleled accuracy of 99%. Moreover, the precision of 100% and recall of 98% further affirm the model’s reliability and robustness in clinical applications.

### 4.3 Strengths and limitations

This study has several strengths that highlight its contribution to the field of medical diagnosis. First, the use of stacked learning models, combined with ADASYN for class imbalance handling and BORUTA for feature selection, enabled the development of a robust and interpretable diagnostic framework for both PCOS and cervical cancer detection. The proposed framework achieved high classification accuracy and recall, outperforming existing methods in detecting both conditions. The application of ADASYN effectively improved the model’s ability to handle imbalanced datasets, making it particularly relevant for real-world medical scenarios where such imbalances are common. Additionally, the use of BORUTA for feature selection reduced the feature space, improving model interpretability and enabling a clearer understanding of the key factors influencing disease detection. Despite these strengths, there are some limitations that should be acknowledged. First, the study was conducted on relatively small datasets for both PCOS and cervical cancer detection, which may limit the generalizability of the findings. Future research should focus on validating the proposed framework on larger, more diverse datasets to ensure its robustness across different populations and demographic groups. Furthermore, while ADASYN was effective in addressing class imbalance, more advanced techniques could be explored in future research to further optimize performance, particularly in highly imbalanced datasets. Additionally, the reliance on BORUTA for feature selection, although it improved model interpretability, may have excluded latent features or interactions that could be important. Future work could investigate more advanced feature selection methods, such as deep learning-based approaches, to capture hidden patterns in the data. Lastly, while ensemble techniques like stacked learning models were beneficial for performance, they are computationally intensive. Future studies may explore lightweight models that maintain high accuracy while being more efficient for real-time applications, especially in resource-constrained clinical environments.

### 4.4 Clinical implications

The results of this study have significant clinical implications for the early detection and diagnosis of both PCOS and cervical cancer. The proposed framework, combining Stacked Learning with ADASYN and BORUTA, not only addresses the common issue of class imbalance in medical datasets but also enhances model performance in terms of accuracy, precision, and recall. In clinical practice, accurate detection of these conditions is critical for timely intervention and personalized treatment. For PCOS, early and accurate diagnosis can lead to better management of symptoms and prevent long-term complications such as infertility, metabolic syndrome, and cardiovascular disease. The high performance of the proposed models, particularly in detecting PCOS cases with imbalanced data, indicates their potential for use in real-world clinical settings where accurate diagnosis is crucial for effective treatment.

Similarly, the accurate classification of cervical cancer is essential for preventing disease progression and improving patient outcomes. The high recall and precision rates achieved by the proposed framework minimize the risk of false negatives, which is vital in ensuring that all cancer cases are detected and treated promptly. This has profound implications for cervical cancer screening programs, where early detection plays a pivotal role in reducing mortality rates. The study demonstrates the potential of advanced machine learning models, combined with feature selection and data balancing techniques, to significantly improve the accuracy and reliability of medical diagnoses. These findings offer valuable insights into the application of artificial intelligence in healthcare, paving the way for more efficient and effective diagnostic tools that can be integrated into clinical practice.

## 5 Conclusion

This paper introduced a framework for the classification of PCOS and cervical cancer, demonstrating promising results with significant implications. By employing an integrated approach that combines stacked learning, the ADAYSN algorithm for data balancing, and the BORUTA technique for feature selection, a classification accuracy of 97% for PCOS diagnosis was achieved. For cervical cancer classification, the framework exhibited exceptional performance, achieving an accuracy of 99%, a recall of 98%, a precision of 100%, and an F1 score of 99%. While these results represented a substantial advancement, it is crucial to validate the robustness and generalizability of the framework through extensive testing on diverse and larger datasets. Future research directions include enhancing the interpretability of the model to gain insights into its decision-making processes and integrating it with established clinical protocols. This will facilitate a better understanding of the complexities associated with PCOS and cervical cancer diagnosis and treatment. The proposed framework demonstrated considerable promise in improving classification accuracy for PCOS and cervical cancer. This potential has the capacity to significantly impact healthcare practices by providing clinicians with a reliable tool for informed decision making, ultimately leading to improved patient outcomes. Continued scholarly investigation in this area is essential for the development of innovative computer-aided diagnostic systems tailored to address the complexities of these medical conditions, effectively.

## Data Availability

The datasets presented in this study can be found in online repositories. The names of the repository/repositories and accession number(s) can be found below: Polycystic ovary syndrome (PCOS), available at: https://www.kaggle.com/datasets/prasoonkottarathil/polycystic-ovary-syndrome-pcos/ (accessed 2023-06-10).
